# Spectroscopic Analyses of the Biofuels-Critical Phytochemical Coniferyl Alcohol and Its Enzyme-Catalyzed Oxidation Products 

**DOI:** 10.3390/molecules14114758

**Published:** 2009-11-23

**Authors:** Komandoor Elayavalli Achyuthan, Paul David Adams, Blake Alexander Simmons, Anup Kumar Singh

**Affiliations:** 1 Joint BioEnergy Institute (JBEI), Emeryville, CA 94550, USA; 2 Biosensors and Nanomaterials Department, Sandia National Laboratories, Albuquerque, NM 87185, USA; 3 Lawrence Berkeley National Laboratory, Berkeley, CA 94720, USA; E-Mail: pdadams@lbl.gov (P.D.A.); 4 Sandia National Laboratories, Livermore, CA 94550, USA; E-Mails: basimmo@sandia.gov (B.A.S.); aksingh@sandia.gov (A.K.S.)

**Keywords:** coniferyl alcohol, absorption spectroscopy, high-throughput screening, monolignols, biofuels

## Abstract

Lignin composition (monolignol types of coniferyl, sinapyl or *p*-coumaryl alcohol) is causally related to biomass recalcitrance. We describe multiwavelength (220, 228, 240, 250, 260, 290, 295, 300, 310 or 320 nm) absorption spectroscopy of coniferyl alcohol and its laccase- or peroxidase-catalyzed products during real time kinetic, *pseudo*-kinetic and endpoint analyses, in optical turn on or turn off modes, under acidic or basic conditions. Reactions in microwell plates and 100 μL volumes demonstrated assay miniaturization and high throughput screening capabilities. Bathochromic and hypsochromic shifts along with hyperchromicity or hypochromicity accompanied enzymatic oxidations by laccase or peroxidase. The limits of detection and quantitation of coniferyl alcohol averaged 2.4 and 7.1 μM respectively, with linear trend lines over 3 to 4 orders of magnitude. Coniferyl alcohol oxidation was evident within 10 minutes or with 0.01 μg/mL laccase and 2 minutes or 0.001 μg/mL peroxidase. Detection limit improved to 1.0 μM coniferyl alcohol with *Km* of 978.7 ± 150.7 μM when examined at 260 nm following 30 minutes oxidation with 1.0 μg/mL laccase. Our assays utilized the intrinsic spectroscopic properties of coniferyl alcohol or its oxidation products for enabling detection, without requiring chemical synthesis or modification of the substrate or product(s). These studies facilitate lignin compositional analyses and augment pretreatment strategies for reducing biomass recalcitrance.

## 1. Introduction

Plant lignin is chiefly composed of three phytochemicals: the monolignols of coniferyl (CA), sinapyl (SA) and *p*-coumaryl (*p*-CA) alcohols. Depending on the species or tissue type, the monolignol content varies [1]. For example, guaiacyl lignin of softwood (Gymnopserms) is principally composed of CA [1,2]. Lignin composition plays a key role in biomass recalcitrance and delignification is a primary challenge facing cost-effective biofuels [3]. Lignin analyses will facilitate plant genetic engineering by changing the monolignol composition [2,4] in order to reduce recalcitrance. Estimating the monolignol type and content will also enable the assignment of appropriate biomass pretreatment strategies to breakdown lignin, reduce recalcitrance and improve saccharification [5].

Toward these goals, we are developing high throughput screening (HTS) strategies for monolignols and their oxidation products, starting with CA. The white rot filamentous fungus *Trametes versicolor* (*T. versicolor*) has a high capacity for degrading lignin [6] either directly through the oxidative, demethylating and demethoxylating activities of phenoloxidative enzymes such as laccases and peroxidases or *via* redox shuttle mediators (RSM) arising from nonspecific free radical mechanisms [7,8,9]. Our choice of CA was also motivated by a desire to explore low cost, nontoxic, high performing RSM. Our prior efforts with *p*-cresol [10,11] as a potential RSM and a report that CA itself was a RSM [12] were additional reasons to focus upon this phytochemical. The *T. versicolor* laccase was selected based on our prior experience with this enzyme [10,11] and further predicated on its high efficiency lignin degradation as well as CA transformation capabilities [6,13].

Published methods for CA analysis included high performance liquid chromatography [14], gas chromatography [15], capillary zone electrophoresis [12] or oxygen consumption [16] requiring sophisticated equipment and/or trained operators. Others have reported nonspecific chemical reactions for colorimetric detection [17,18] that are unsuited for HTS of monolignols (11) based upon design considerations described previously [19]. Coniferyl alcohol was also monitored by absorption at 214 nm [12], 260 to 265 nm [20,21,22,23,24] or 296 nm [25], and oxidized CA at 400 nm [26,27,28]. However, the rationale for these wavelengths was not explicit or was based upon the formation of coniferaldehyde that might not be the only end product of CA enzymatic transformation. These assays are also not HTS-compatible due to their large (1 to 3 mL) reaction volumes [20,21,22,23,24,25,26,29].

We therefore undertook a systematic examination of the intrinsic spectroscopic properties of unmodified CA as well its oxidation products following laccase and peroxidase catalysis. Our assays are HTS-compatible, requiring no chemical synthesis, labeling or modifications to the substrate or to the product. The assays employ only the natural phytochemical CA and not an artificial substrate tethered to a reporter molecule. Our assays offer a choice of operations including real time kinetic, *pseudo*-kinetic or endpoint mode in either optical turn on or turn off signaling. The assays accommodate acidic or basic pH. A broad range of wavelengths supplies a menu of interrogation options. Eventually, we hope to develop a portable, field deployable, multiplexed, orthogonal HTS sensor [11] capable of remote/standoff detection of biomass monolignols and facilitate lignin compositional analyses.

## 2. Results and Discussion

### 2.1. Preliminary Absorption Analysis of CA

Citrate buffer of pH 4.5 was previously established as being optimum for the laccase-catalyzed oxidation of the phenolic substrate *p*-cresol [10]. We therefore employed the same buffer for CA oxidation, since the pH optimum is similar with different phenolic substrates for a particular laccase, in this case, the *T. versicolor* enzyme [10,11]. If laccase from a different source was substituted, then a re-examination of the pH-activity profile might be warranted. Although there was a 2-fold increase in laccase oxidation of *p*-cresol by elevating the temperature to 37 °C from 25 °C [10], we chose to carry out CA oxidation at 25 °C in order to make our biosensor simple and portable [11]. All reactions were conducted in 96-well UV-transparent clear microplates in 100 μL volume resulting in less waste and greater sample conservation as well as being HTS-compatible [19]. This is in contrast to the reaction volumes of 1 to 3 mL used previously for detecting/quantifying CA or its enzymatic products [20,21,22,23,24,25,26,29]. We also found that either 100 mM NaOH or 250 mM KOH was optimal for maximum signal strength and either base could be used. These observations are in accordance with our *p*-cresol results [10] and hinted at the overall similar ionization processes of the two chromophores (*p*-cresol and CA) in basic pH. The pKa of CA is 9.5 requiring >pH 9 conditions in order to deprotonate the phenolic groups, similar to *p*-cresol [30]. There were however significant differences in the spectroscopic behavior of the two analytes, especially following enzymatic oxidation as discussed in this paper.

### 2.2. Absorption Spectroscopic Profiles of Unmodified CA

**Figure 1:**
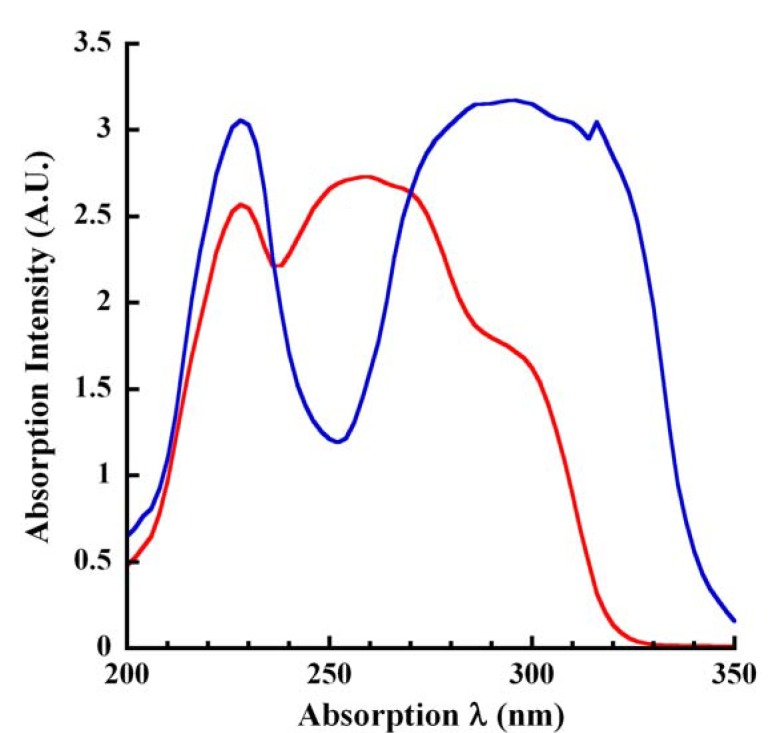
Absorption spectra of unmodified CA. The absorption of CA (100 μL *Vt*; 250 μM final) was measured at ~25 °C under acidic (citrate buffer, pH 4.5; red tracing) and basic (250 mM KOH; blue tracing) pH conditions in a 96-well UV-transparent microplate. Absorption was scanned over the wavelength range of 200 to 350 nm in 2 nm intervals. Absorption profiles were corrected for background from the vehicle (pH 4.5 buffer or 250 mM KOH).

We next undertook a systematic examination of the absorption profiles of unmodified CA, a natural lignin phytochemical in pH 4.5 citrate buffer and in base (Figure 1). When the experiment was repeated after an interval of three months, nearly identical profiles were generated, thus confirming the repeatability and reproducibility of our spectroscopic assays. The data also demonstrated the stability of CA since the compound was described as air- and light-sensitive in the certificate of analysis furnished by the vendors. Furthermore, CA from two different sources (Sigma-Aldrich, Alfa Aesar) displayed similar but *not* identical absorption profiles. The bathochromic shifting of the larger absorption peak in base relative to the absorption in pH 4.5 was predictable due to the alcohol becoming deprotonated in base yielding the corresponding anion [30]. The absorption spectra in pH 4.5 and in base were however similar over the wavelengths of 200 to 240 nm (Figure 1). In pH 4.5 buffer, λ_max1_ was 228 nm, λ_max2_ was 258 nm to 260 nm, λ_shoulder_ was 295 nm and λ_min_ was 236 nm. In base, λ_max1_ was also at 228 nm but λ_max2_ shifted to 294 to 296 nm; ~30 nm bathochromicity relative to the pH 4.5 peak absorption. Likewise, the λ_min_ also red-shifted to 252 nm and the λ_shoulder_ seen in pH 4.5 absorption profile disappeared in base (Figure 1). Our data are similar to the 265 nm λ_max_ at pH 7.0 and the bathochromic shift to 290 nm in basic pH reported previously [31]. However, this report did not describe the λ_max1_ at 228 nm seen under both acidic and basic pH conditions (Figure 1).

### 2.3. Isosbestic Points of Unmodified CA

An isosbestic point is a wavelength at which two or more compounds have the same molar absorptivity. In other words, it represents wavelengths at which the absorbance of two substances, one of which can be converted into the other, is the same. Using the same concentration of CA, absorption spectra were generated under acidic and basic pH conditions resulting in the two profiles intersecting at 236 nm and 270 nm; an isosbestic plot (Figure 1). Thus, two isosbestic points were concluded at these wavelengths. This occurred because the nonionized and ionized forms of CA absorbed light at these wavelengths to the same extent and the analyte concentration remained constant. Isosbestic points have applications as reference points for reaction rates and in quality assurance since the isosbestic point does not depend on the concentration of the analyte and therefore becomes a reliable reference. One of the isosbestic points determined here was close to the 280 nm reported previously [20].

### 2.4. Sensitivity of Unmodified CA

**Figure 2:**
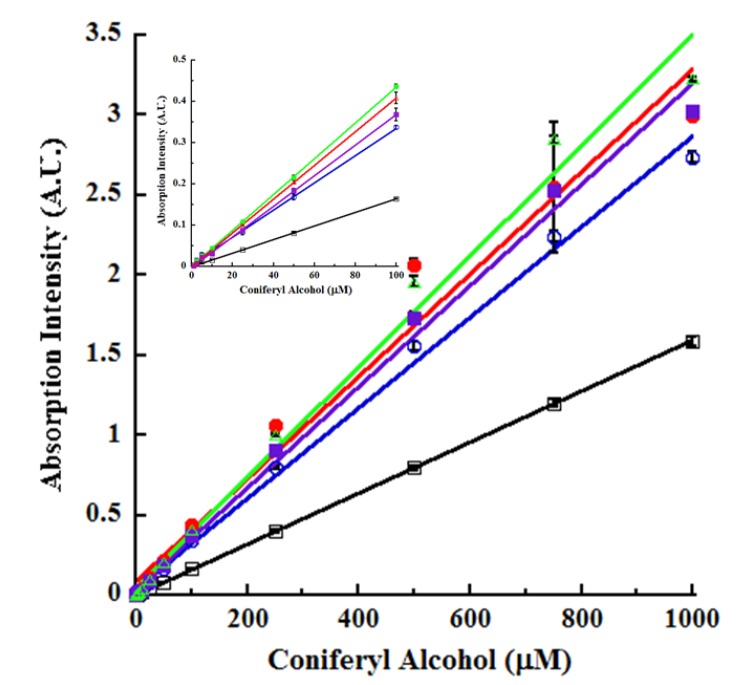
Sensitivity of unmodified CA detection. Increasing and indicated concentrations of unmodified CA (100 μL) were dispensed in 96-well UV-transparent microplate. Absorption from CA was measured under the following reaction conditions: pH 4.5, 228 nm (open circles; blue); pH 4.5, 260 nm (closed circles; red); pH 4.5, 295 nm (open squares; black); 250 mM KOH, 228 nm (closed squares; purple); and 250mM KOH, 295 nm (open triangles; green). The linearity values of the correlation of coefficients (*r^2^*) for the various tracings are provided in Table 1. The inset shows the 0 to 100 μM CA dose-response tracings. All other conditions were as in Figure 1.

Utilizing the absorption wavelengths identified above, we next quantified unmodified CA. The data are shown in Figure 2.

We detected CA under acidic (pH 4.5) conditions using the absorption wavelengths of 228, 260 and 295 nm. We also quantitated CA under basic (250 mM KOH) conditions using absorption wavelengths of 228 and 295 nm. From a reference to the tracings of Figure 1, it is apparent that these wavelengths represented the λ_max_ or λ_shoulder_ for CA under acidic conditions and λ_max_ in basic medium, and therefore well suited for assaying CA. The limits of detection (LOD) and quantitation (LOQ) for native, unmodified CA averaged 2.4 and 7.1 μM, respectively (Table 1). Absorption spectroscopy offered excellent dynamic range by being linear over three orders of magnitude between 1 to 1000 μM CA, at all the wavelengths examined, under both acidic and basic conditions (Figure 2). The absorption wavelengths of 260 nm (pH 4.5) and 295 nm (pH 4.5 and KOH) yielded even superior dose-response. The trend line of 295 nm absorption (pH 4.5) was suggestive of linearity over four orders of magnitude up to 10,000 μM CA (Figure 2). Due to solubility limitations of CA as well as being irrelevant to physiological concentrations [29], measurements of CA were not extended to 10 mM. Linearity of the dose-response tracings was also uniformly excellent with the correlation of coefficient (*r^2^*) exceeding 0.97 in all cases (Table 1).

The sensitivity of our CA’s intrinsic absorption spectroscopic assay compared favorably against a chemical assay using Gibbs reagent [18]. In the latter assay, 50 μM CA was quantified. However, it was not clear whether this represented the LOD for CA [18]. Gibbs reagent reacts with phenols and is not specific for CA. Furthermore, the same wavelength (562 nm) was used to detect CA, SA and ferulic acid, thereby minimizing assay specificity [18]. A different CA assay utilizing a reaction with Fast Blue RR did not report on the detection limits [17]. Sensitivities of 0.1 to 0.4 μM CA were reported using laccase immobilized on graphite electrodes followed by electrochemical measurements [32,33]. The sensitivity of lignin model compounds by HPLC was 20 to 500ng [34]. These values are within range for CA LOD (2.4 μM = 43 ng/100 μL *Vt*). Overall, CA detection limits were similar to the 1 to 10 μM reported for another potential RSM, namely, *p*-cresol [10,11].

**Table 1:** Unmodified CA limits of detection (LOD) and quantitation (LOQ).

Reaction pH	wavelength (λ)	LOD ( μM)	LOD (ppb)	LOQ ( μM)	LOQ (ppm)	Linearity (*r^2^*)
4.5	228 nm	2.5	435	7.5	1.3	0.99589
4.5	260 nm	1.8	313	5.4	1.0	0.97770
4.5	295 nm	2.5	435	7.5	1.3	0.99998
^*^KOH	228 nm	2.5	435	7.5	1.3	0.99434
^*^KOH	295 nm	2.5	435	7.5	1.3	0.98838

^*^ KOH concentration was 250 mM final. The legend to Figure 2 contains additional experimental details for the data shown in this Table.

### 2.5. Advantages of Our CA Assays

A choice of several absorption wavelengths under acidic and basic reaction conditions offers multiple interrogation opportunities for unknown samples, thus improving sensitivity and specificity [19]. Furthermore, our data offers an explanation for the choice of wavelengths employed to detect and quantify native, unmodified CA. Previously, *p*-CA and SA were measured using the absorption wavelengths of 260 and 270 nm [21]. These wavelengths are close to one of the major absorption peaks of CA and likely to interfere in the measurements of different monolignols. On the other hand, the flexibility of the several absorption wavelengths identified in this paper offers a way to eliminate or minimize these interferences.

Another advantage of the current assay is to monitor the activity of enzymes capable of generating or liberating CA or oxidizing CA. For example, eugenol and vanillyl alcohol oxidases convert eugenol to CA [25,31]. This reaction may be conveniently monitored using the various absorption wavelengths of Figure 1, Figure 2 under acidic or basic conditions in real time kinetic or endpoint mode of these oxidases. Furthermore, eugenol/vanillyl alcohol oxidase catalysis might be terminated at predefined, timed intervals using base (100 mM NaOH or 250 mM KOH) and then the absorbance of the CA generated may be measured under *pseudo*-kinetic mode.

Another important class of enzymes for biofuels is the β-glucosidase, especially those involved in hydrolyzing the lignin precursor coniferin (4-O-β-D-glucopyranoside of CA) and liberating CA [17,35,36]. In β-glucosidase assays, the aglycone was not measured [35,36]. Instead, the released sugar was quantified using HPLC [35] or reaction with Fast Blue [17], both time-intensive procedures. Alternately, the released aglycone may be quantified by reaction with the Gibbs reagent [18]. Neither Fast Blue nor Gibbs reagent is specific for CA. Synthetic substrates employing an enzyme-cleavable chromophore or fluoropore while useful for rapid screening provide little information regarding the specificity of the enzyme for natural phytochemical substrates such as coniferin and have little relevance to the physiological reactions of the plant cell walls. The situation becomes acute with a promiscuous enzyme such as β-glucosidase capable of hydrolyzing several different glucosides, releasing various types of sugars [35,36]. Using the menu of spectroscopic interrogation wavelengths for native, unmodified CA (Figure 1, Figure 2), we provide a HTS-alternative for β-glucosidase activity toward coniferin, by targeting the aglycone.

Other advantages of our CA assay include, sensitivity, speed, multi-wavelength interrogations, optical "turn on" or "turn off" signaling, with or without enzymatic catalysis, detecting under real-time kinetic, *pseudo*-kinetic or endpoint modes in acidic or basic milieu using minaturized reaction volumes, HTS-capability, and finally, monitoring CA without resorting to chemical modification, but instead utilizing the CA's intrinsic spectroscopic properties. Spectroscopy of enzymatically-oxidized CA is described below. We are leveraging this suite of capabilities for developing a field-portable analytical sensor for lignin compositional analyses of biomass, in order to reduce recalcitrance, through a rational selection amongst pretreatment strategies, for producing cost-effective biofuels.

### 2.6. Kinetics of Laccase-Oxidized CA

We next investigated laccase oxidation of CA by monitoring the absorption spectroscopic changes utilizing the wavelengths identified in Figure 1, Figure 2. These measurements were conducted using various concentrations of the enzyme in real time kinetic mode (pH 4.5 reactions), with the absorption spectra being collected at 13, 24, 36, 47, and 57 minutes after initiating laccase catalysis. At 57 minutes, the enzymatic reaction was terminated by the addition of 250mM KOH (resulting in the 60 minutes endpoint) and the absorption spectra were collected in basic pH. In view of the large number of kinetic absorption profiles collected, only those representing the reactions at 13 and 57 minutes are shown in Figure 3A, Figure 3B. As expected, the absorption profiles at 24, 36 and 47 minutes were progressively intermediate to the profiles shown for 13 and 57 minutes.

An examination of these tracings highlighted the following observations and conclusions. With increasing concentrations of laccase or prolonged catalysis time, the absorption at 220 nm became hypochromic as seen by the drop in intensity from 2.3847 A.U. at 13 minutes of catalysis (Figure 3A) to 1.3874 A.U. at 57 minutes with 2.0 μg/mL laccase (Figure 3B). A similar hypochromicity was also noted at 220 nm in an laccase concentration-dependent fashion. These trends were also reflected progressively in the absorption profiles collected at the intermediate reaction periods of 24, 36 and 47 minutes (data not shown). A similar hypochromicity was seen at 240 nm. The next major observation was the marked broadening and 14 nm bathochromicity (to 274 nm) of the relatively sharp absorption peak at 260 nm in pH 4.5 buffer, as a consequence of oxidation time and/or increasing concentrations of laccase (compare Figure 3A, Figure 3B). With 2 μg/mL laccase at 57 minutes catalysis, the 260 nm peak disappeared resulting in a nearly flat line around this absorption region (Figure 3B). A further consequence of laccase oxidation was the hypochromicity surrounding the 260-274 nm absorption wavelengths similar to the hypochromicity of 220 to 240 nm. There was also the appearance of a new “shoulder” peak between 290 and 310 nm with increasing reaction time and/or increasing laccase concentration accompanied by hyperchromicity at 320 nm (Figure 3A, Figure 3B). As before, these features were progressive for the intermediate reactions of 24, 36 and 47 minutes.

**Figure 3A:**
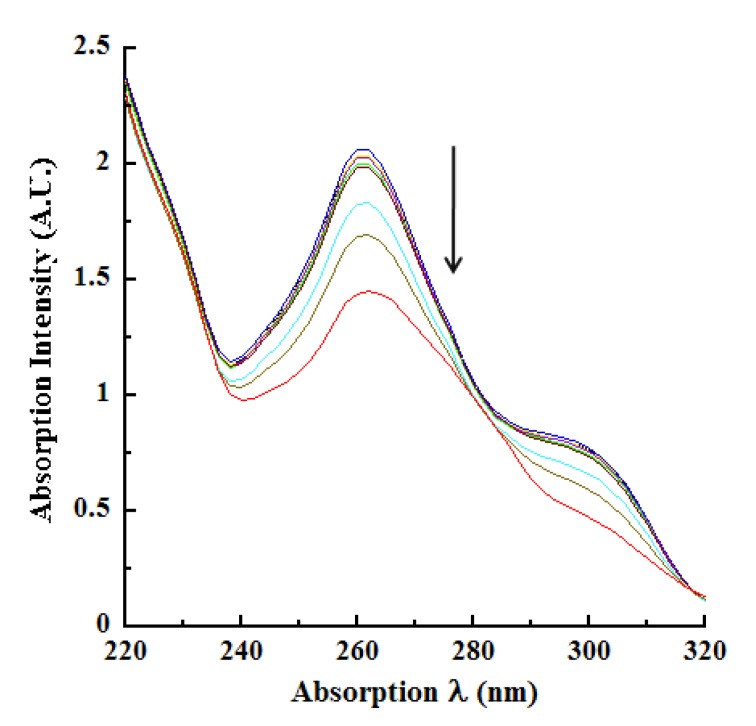
Laccase oxidation of CA at 13 minutes. Increasing concentrations of *T. versicolor* laccase was reacted with 500 μM CA for 13 minutes at pH 4.5 in real time kinetic mode. The changes in absorption intensities consequent to enzyme catalysis were monitored by scanning between 220 to 320 nm in 2 nm intervals. The various tracings with progressively decreasing absorption intensities, indicated by the downward pointing arrow, represented reactions in the absence of laccase (0 μg/mL; black tracing) as well as increasing concentrations ( μg/mL) of laccase as follows: 0.01 (yellow), 0.025 (blue), 0.05 (purple), 0.1 (green), 0.25 (magenta), 0.5 (aqua), 1.0 (brown) and 2.0 (red). All other conditions are as described in the legend to Figure 1.

**Figure 3B:**
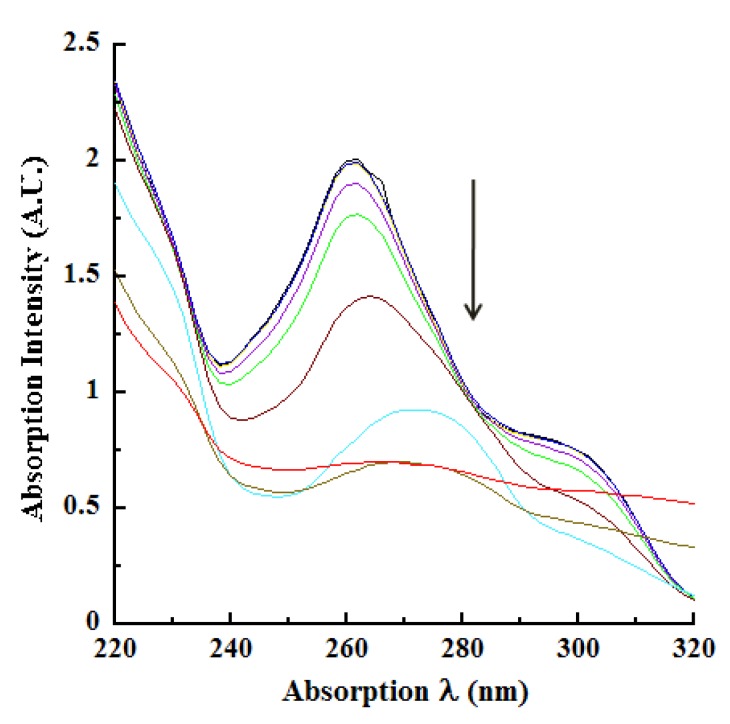
Laccase oxidation of CA at 57 minutes. Laccase-catalyzed oxidation of CA was monitored at 57 minutes in kinetic mode. All other conditions are as described in the legend to Figure 3A. The nine tracings representing the reactions in the absence of enzyme (0 μg/mL; black tracing) and the eight concentrations of laccase listed in Figure 3A (indicated by the downward pointing arrow) are better distinguished in Figure 3B due to the reaction progress. Laccase oxidation of CA at 57 minutes. Laccase-catalyzed oxidation of CA was monitored at 57 minutes in kinetic mode. All other conditions are as described in the legend to Figure 3A. The nine tracings representing the reactions in the absence of enzyme (0 μg/mL; black tracing) and the eight concentrations of laccase listed in Figure 3A (indicated by the downward pointing arrow) are better distinguished in Figure 3B due to the reaction progress.

### 2.7. Endpoint Absorption Profiles of Laccase-Oxidized CA

**Figure 4:**
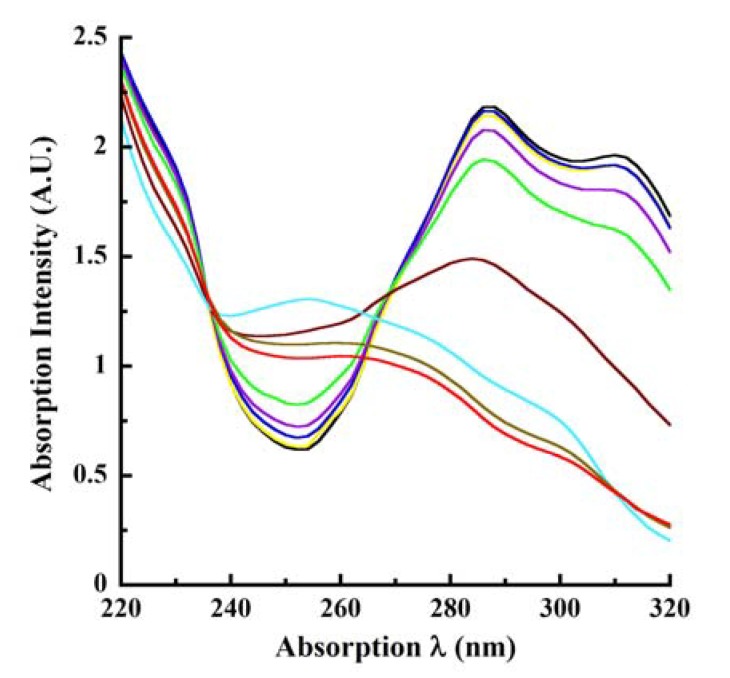
Endpoint laccase oxidation of CA. The enzymatic reactions of Figure 3A, Figure 3B were terminated with base (250 mM KOH). There was three minutes lag between the kinetic scans at 57 minutes and the addition of base resulting in the 60 minutes endpoint measurement. All other conditions were as described for Figure 1A, Figure 3A and Figure 3B. The tracings represent reactions in the absence of laccase (0 μg/mL; black) and the eight concentrations of laccase listed in Figure 3A.

The pH 4.5 reaction mixtures (above) were basified using 250 mM KOH, terminating laccase catalysis at 60 minutes. Reaction mixtures were re-scanned by absorption spectroscopy and yielded dramatically different profiles (Figure 4). First, the magnitude of hypochromicity at 220 nm absorption (pH 4.5; Figure 3B) became severely compressed in base, and thus eliminated this wavelength for quantifying CA oxidation under basic conditions. More dramatic was the peak inversion resulting in the formation of an absorption trough and hypsochromicity to 250 nm (from 260 nm) along with hyperchromicity following laccase catalysis (Figure 4). For example, the 250 nm absorption of CA in the absence of laccase was 0.6305 A.U. in base. In the presence of 0.5 μg/mL laccase, the absorption at this wavelength increased to 1.2928 A.U. (Figure 4). Thus the absorption profiles around 250 to 260 nm are inverted mirror images at pH 4.5 and in base. A final noteworthy feature of the absorption profiles under basic conditions was the appearance of a new and broad “shoulder” peak between 280 and 320 nm accompanied by hypochromicity. Thus, the absorption profiles in base yielded three new interrogation wavelengths of 288, 310 and 320 nm. Similar to the pH 4.5 reactions, these spectroscopic changes were detected progressively during the intermediate catalytic periods of 24, 36 and 47 minutes.

### 2.8. Difference Spectroscopy of Unmodified and Laccase-Oxidized CA

**Figure 5:**
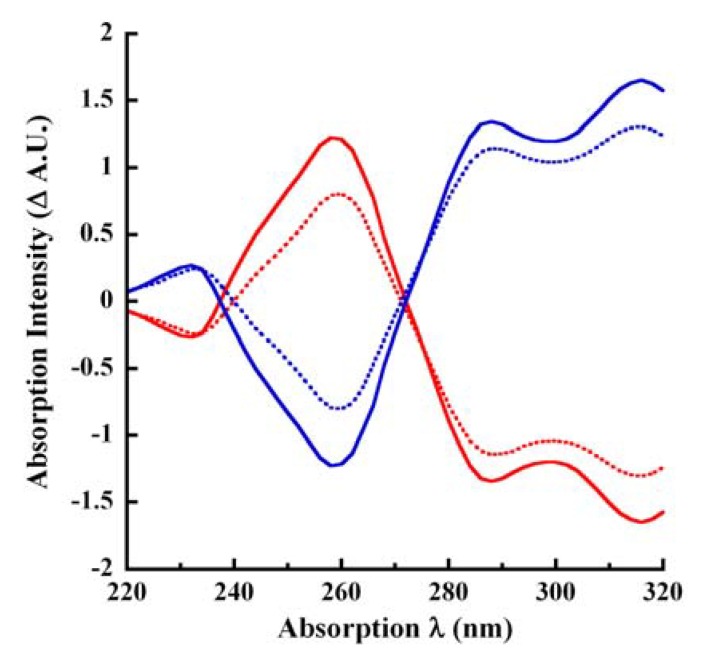
Difference spectroscopy of CA. Subtractive absorption profiles of unmodified CA in pH 4.5 buffer or in 250 mM KOH and profiles of 0.1 μg/mL laccase-oxidized CA in pH 4.5 buffer (57 minutes) or in 250 mM KOH (60 minutes) are shown here. The solid red tracing represents the absorption of unmodified CA measured in pH 4.5 buffer subtracted from the absorption in 250 mM KOH. The solid blue tracing represents the absorption of unmodified CA taken in 250 mM KOH subtracted from its absorption in pH 4.5 buffer. The dotted red tracing is the profile of laccase-oxidized CA in pH 4.5 buffer subtracted from its absorption in 250 mM KOH. Finally, the dotted blue tracing is the absorption of laccase-oxidized CA reactions terminated with 250 mM KOH subtracted from its absorption in pH 4.5 buffer.

The UV-Vis difference spectra facilitate the identification of multicomponent mixtures [37]. In alkaline solution, the phenolic hydroxyl groups are ionized and the absorption spectrum reflects this process by red-shifting to longer wavelengths. In order to obtain an ionization difference spectrum (Δ_ε__i_-spectrum), the absorption intensities at pH 4.5 were subtracted from the intensities in KOH and *vice versa*. Coniferyl alcohol is an aromatic compound containing hydroxyl auxochromic group and its difference spectra showed both positive and negative absorptivity that are related to the electronic transitions and the degree of protonation [37]. In order to tease out suitable interrogation wavelengths, we examined the difference spectroscopy of unmodified and laccase-oxidized CA under acidic (pH 4.5) and basic (250 mM KOH) conditions (Figure 5). The bathochromic spectral shifts in base for unmodified CA (Figure 1, Figure 5) are reminiscent of the work of Fraaije *et al.* [31].

### 2.9. Dose-Response Tracings for Laccase-Oxidized CA

Based upon the above data, we decided upon the following absorption wavelengths for interrogating laccase-catalyzed oxidation of CA: 220, 240, 260, 295, 300 and 320 nm in kinetic mode at pH 4.5 (Figure 6A) along with 250, 290, 310 and 320 nm in endpoint mode with 250 mM KOH (Figure 6B). It should be noted that the absorption changes reflected at these various interrogation wavelengths included both hyperchromicity (250 nm in KOH and 320 nm in pH 4.5 buffer) calculated as signal minus background (S−B) and hypochromicity (rest of the wavelengths) calculated as background minus signal (B−S). The data in Figure 6 show the dose-response behavior for laccase catalysis with the velocity saturating in both kinetic 57 minutes (Figure 6A) and endpoint 60 minutes reactions (Figure 6B). Both reactions saturated between 0.5 and 1 μg/mL laccase. The highest Δ A.U. was noted at 260 nm (pH 4.5 kinetic reactions), whereas nearly equal and high Δ A.U. values were observed at 288, 310 and 320 nm in endpoint mode. These reactions could also be monitored in a *pseudo*-kinetic mode by stopping the reaction at predetermined timed intervals using base.

**Figure 6A:**
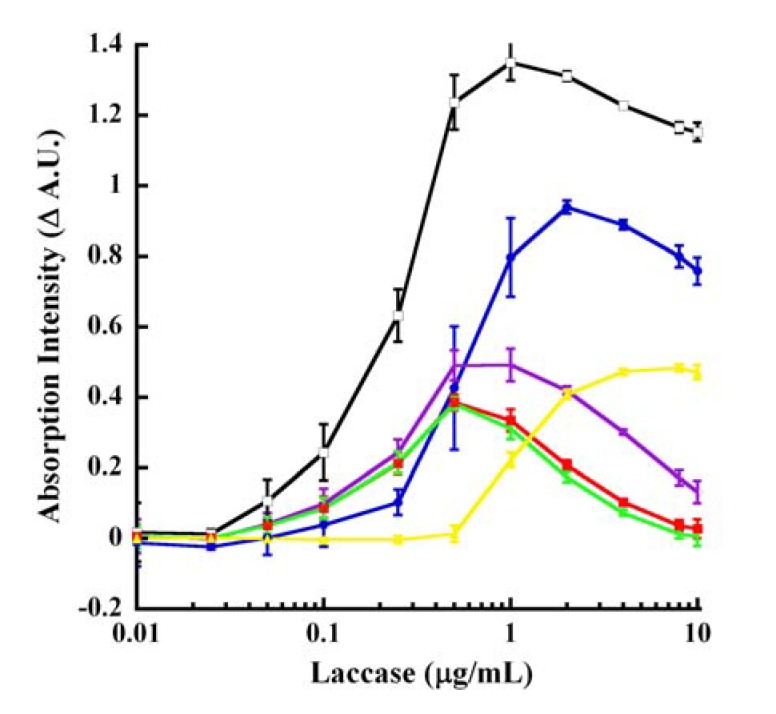
Laccase dose-response at 57 minutes of CA oxidation. Increasing and indicated concentrations of laccase were reacted with 500 ¼M CA in pH 4.5 buffer for 57 minutes as described in the legend to Figure 3B. The various colored tracings represent the following absorption wavelengths: 220 nm (blue), 240 nm (purple), 260 nm (black), 295 nm (red), 300 nm (green) and 320 nm (yellow). All tracings represent the difference between the absorption intensities of the reactions without enzyme subtracted from the absorption of the reactions in the presence of laccase (B−S), except for 320 nm which is the discrimination between the signal (with laccase) and the background (without laccase enzyme) (S−B).

**Figure 6B:**
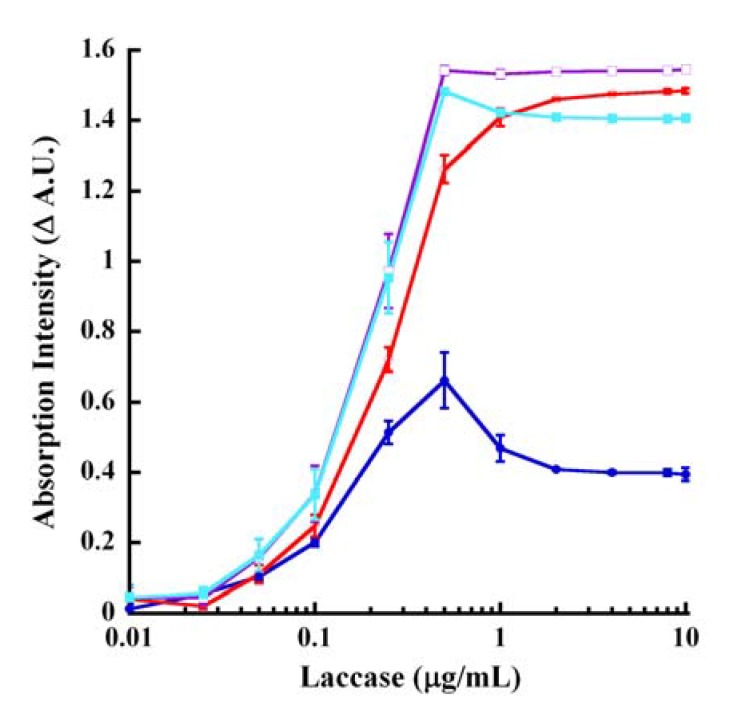
Endpoint laccase dose-response of CA oxidation. Laccase reactions (from Figure 6A) were terminated at 60 minutes with 250mM KOH. The tracings represent the absorption wavelengths of 250 nm (blue), 290 nm (red), 310 nm (purple) and 320 nm (aqua). All tracings represent the difference between the absorption intensities of the reactions without enzyme subtracted from the absorption intensities of reactions in the presence of laccase (B−S), except for 250 nm which is the discrimination between the signal (with laccase) and the background (without laccase) (S−B).

### 2.10. Progress Curves for Laccase-Oxidized CA

**Figure 7:**
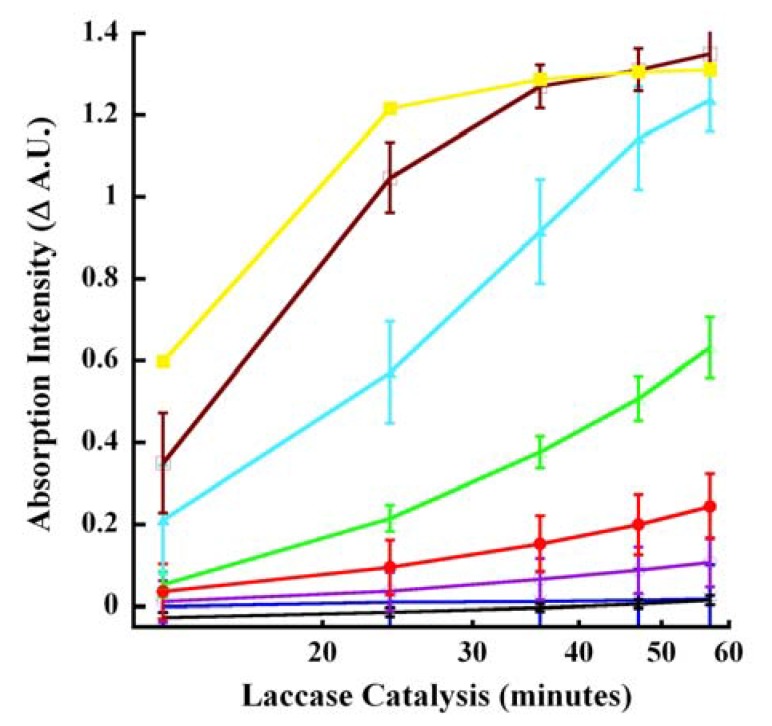
Progress curves for laccase oxidation of CA. Increasing concentrations of laccase were reacted with 500 μM CA for the indicated time-periods. The absorption at 260 nm was measured and the difference in the intensities between reactions without the enzyme and with the enzyme was recorded (B−S). Reactions were carried out in kinetic mode (citrate buffer, pH 4.5). The various colored tracings represent the following concentrations of laccase ( μg/mL): 0.01 (black), 0.025 (blue), 0.05 (purple), 0.10 (red), 0.25 (green), 0.50 (aqua), 1.00 (magenta) and 2.00 (yellow). The linearity values of the correlation of coefficient (*r^2^*) for 0.05, 0.1, 0.25 and 0.5 μg/mL laccase were 0.99783, 0.99789, 0.99829 and 0.96606, respectively.

We identified 260 nm as a major interrogation wavelength for monitoring laccase-catalyzed oxidation of CA in kinetic mode even though dose-response tracings of varying degrees of linearity were also obtained at 220, 240, 295, 300 and 320 nm. Progress curves at 260 nm are shown in Figure 7.

Predictably, signal discrimination (hypochromicity) became magnified between catalysis times of 13 and 57 minutes. Excellent linearity (*r^2^* ≥ 0.97) was observed especially over an order of magnitude of laccase concentrations 0.05 and 0.5 μg/mL (Figure 7). The lower limits of laccase detected using CA as substrate was 10ng/mL enzyme. Depending on the enzyme concentration, CA oxidation could be detected within 10 minutes. This was in contrast to the extended reaction periods used previously, some lasting up to 4 hours [13].

Our data are in agreement with previous reports [20,21,22,23,24,38] identifying 260 to 265 nm as a good region for selecting wavelengths for monitoring CA oxidation even though all these reports used plant peroxidases instead of the fungal laccase employed in the present study. This suggested a similar reaction mechanism for CA oxidation yielding the same or similar oxidation products regardless of laccase or peroxidase catalysis. We amplified this finding by identifying several additional wavelengths for monitoring laccase catalyzed CA oxidation in real time kinetic, *pseudo*-kinetic or endpoint mode under acidic or basic condition. Data generated from multiple interrogation wavelengths with or without laccase catalysis offer flexibility and facilitate ratiometric analyses, an important statistical tool for improving assay specificity [19] by minimizing the effects of interfering compounds during HTS. Another advantage of multiwavelength interrogation is the elimination of the overlap of the absorption at 260 nm for both CA and *p*-CA oxidation products [21] that introduces uncertainty.

### 2.11. Affinity Constant of CA for Laccase

**Figure 8:**
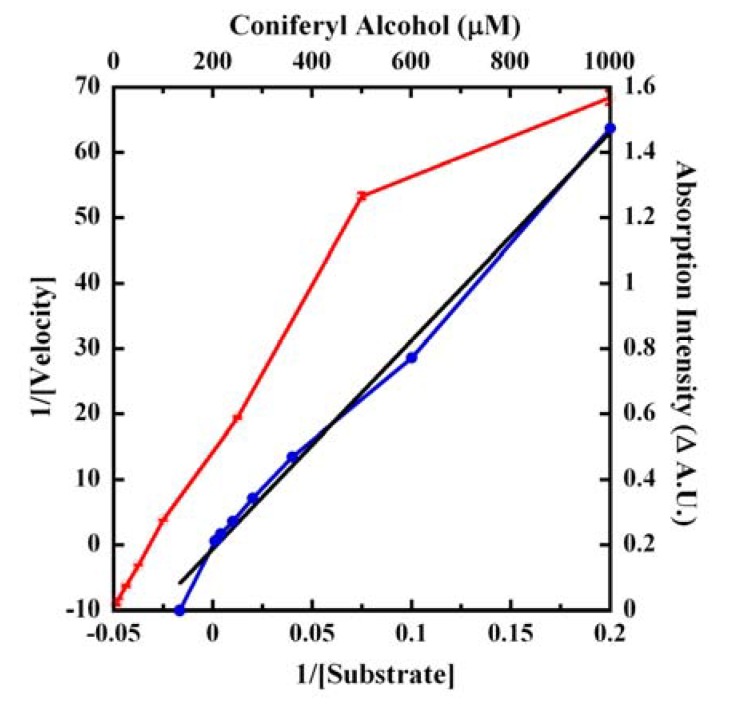
Affinity of CA for laccase. Increasing concentrations of CA were reacted with 1.0 μg/mL laccase for 30 minutes at 25 °C and the absorption at 260 nm was monitored. The open circles (red tracing) represents the Δ A.U. of (B−S) (right ordinate) plotted as a function of CA concentrations (top abscissa). The Lineweaver-Burk transformation of this data is shown as the blue tracing (closed circles) corresponding to the lower abscissa (1/Substrate) and the left ordinate (1/Velocity). The linear curve fit (black tracing) of the transformation is shown along the same axes and the *r^2^* was 0.99192.

We next determined the affinity constant (*Km*) of CA for the laccase (Figure 8).

Since 260 nm gave the highest signal discrimination (Δ A.U.) (Figure 6, Figure 7), this wavelength was chosen for CA affinity calculations in kinetic mode. Similar to *p*-cresol [10], laccase activity increased linearly up to 0.5mM and then the velocity saturated at 1mM CA resulting in the rectangular hyperbola of Figure 8. The catalysis proceeded *via* first order at low concentrations of CA and became mixed at intermediate to higher concentrations of substrate and finally reached zero order rate at high (1mM) concentrations of CA leading to saturation of the velocity. The 260 nm absorption (B−S) of 1.4 to 1.6 Δ A.U. seen under similar reaction conditions for three separate data sets of Figure 6, Figure 7, Figure 8 testified to the repeatability and reproducibility of our assays. A linear (*r^2^*= 0.99192) transformation of the data using Lineweaver-Burk calculations (Figure 8) yielded an affinity constant (*Km*) of 978.7 ± 150.7 μM, representing an error of ~15.4% that was considered acceptable. The CA detection limit improved to 1.0 μM with 1.0 μg/mL laccase catalysis for 30 minutes when the reaction was monitored at 260 nm in kinetic mode. A similar improvement in the detection limits was noted for *p*-cresol following laccase-catalyzed oxidation [10,11]. The *Km* for CA was also in the same range relative to the *Km* of 2.2 mM calculated for *p*-cresol [10], suggesting that the *Trametes versicolor* laccase oxidizes both phenolic substrates with approximately equal affinity.

### 2.12. Peroxidase-Catalyzed Endpoint Oxidation of CA

**Figure 9:**
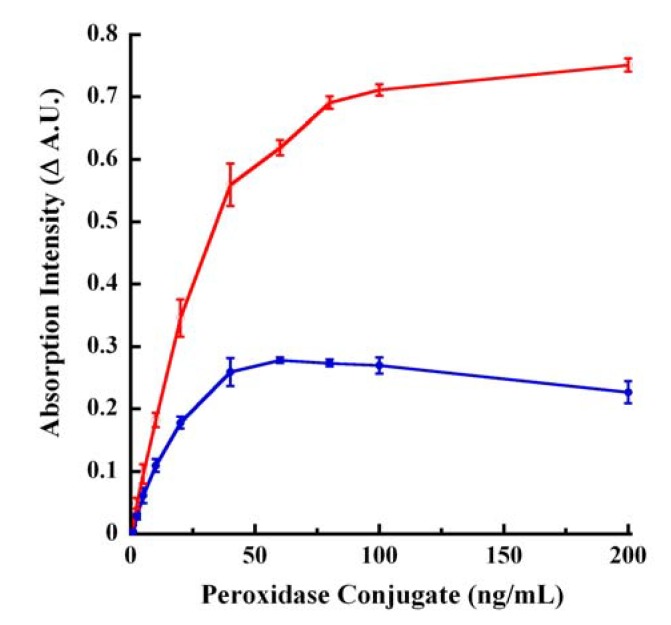
Peroxidase dose-response tracings for CA endpoint mode oxidation. Increasing and indicated concentrations of peroxidase conjugate were reacted with CA for 30 minutes at ~25 °C in 96-well UV-Vis microplate. The reaction was stopped by adding 100 mM NaOH. The red tracing represents Δ A.U. obtained from B−S absorption at 295 nm. The blue tracing represents Δ A.U. obtained from S−B absorption values of 260 nm.

We determined the reactivity of CA as a substrate for a different enzyme, namely peroxidase. Similar to laccase, peroxidase oxidation of CA resulted in either quench or increase of the absorption in base depending on the interrogation wavelength and were monitored in endpoint mode (100mM NaOH) using the wavelengths of 260 and 295 nm. The twin wavelengths were chosen in order to simplify the assay and data analyses and for the reasons that these wavelengths represented Δ A.U. by both S−B (260 nm) and B−S (295 nm) as well as being the low and high end of the absorption scale for laccase oxidation of CA in endpoint mode (Figure 6B). The dose-response tracings of Figure 9 demonstrated a sensitivity of 0.001 μg/mL enzyme. The catalysis was linear up to ~50 ng/mL and saturated thereafter. Similar to laccase oxidation of CA, the Δ A.U. was greater when monitored at 295 nm relative to 260 nm (compare the tracings of Figure 6B, Figure 9).

### 2.13. Kinetics of CA Oxidation Using Peroxidase

Similar to laccase, peroxidase oxidation of CA also resulted in absorption quench at pH 4.5 with the quench increasing with increasing reaction time or enzyme concentration. These changes were monitored in kinetic mode (pH 4.5) using the wavelengths of 260 and 295 nm. Analogous to endpoint measurements, the twin wavelengths were chosen in order to simplify the assay and data analyses and for the reasons that these wavelengths represented Δ A.U. obtained from calculations of B−S as well as being the low and high end absorptions for CA oxidation (Figure 6A). Since both wavelengths showed absorption quench, we used the tracings as a graphical exemplar of our data analyses. Thus, in Figure 10A, we profiled the tracings as progressively decreasing “raw” absorption intensities at 260 nm. By contrast, in Figure 10B, we depicted progressively increasing B−S values at 295 nm. Consequently, in Figure 10B, the negative control black tracings of without peroxidase and without H_2_O_2_ are not shown, since these values were used during the B−S Δ A.U. transformation of the data. Collectively, the data of Figure 10A, Figure 10B show fast reaction kinetics of peroxidase-catalyzed oxidation of CA taking place measurably within 60 to 120 seconds.

**Figure 10A:**
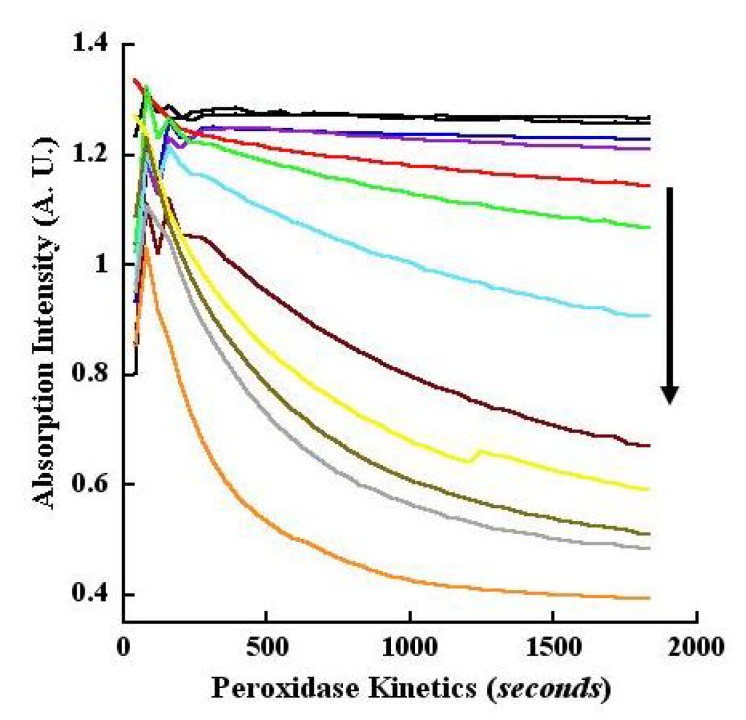
Kinetics of peroxidase oxidation of CA monitored at 260 nm. Increasing concentrations of peroxidase-catalyzed oxidation of CA was monitored at 260 nm in kinetic mode (pH 4.5 buffer). Absorption changes over time and with different concentrations of the enzyme are represented by the various colored tracings with progressively decreasing intensities as indicated by the downward pointing arrow. The concentrations (ng/mL) of the peroxidase were: 1.0 (blue), 2.5 (purple), 5 (red), 10 (green), 20 (aqua), 40 (magenta), 60 (yellow), 80 (brown), 100 (gray) and 200 (orange). The top two black tracings that are nearly parallel to the abscissa represent control reactions without enzyme and without H_2_O_2_, respectively.

Finally, we present the B−S calculated Δ A.U. for 295 nm with *progressively increasing* values with respect to catalysis time and enzyme concentrations (Figure 10B). The kinetic dose-response tracings revealed that CA oxidation took place within 1 to 2 minutes depending on the enzyme concentration. Depending on the reaction time, we observed a sensitivity of 0.001 μg/mL for the peroxidase in the kinetic and endpoint modes (Figure 9, Figure 10). Except at the low end of enzyme concentrations (10 to 20 ng/mL), CA oxidation saturated within 30 minutes (Figure 10A, Figure 10B). Similar to laccase catalysis, the Δ A.U. was greater when monitored at 260 nm relative to 295 nm in the kinetic mode for peroxidase (compare Figure 6, Figure 9, Figure 10). Taken together, the data demonstrated that CA was oxidized similarly by laccase and peroxidase, perhaps yielding the same or similar products, suggesting that the two enzymes behaved promiscuously toward CA.

**Figure 10B:**
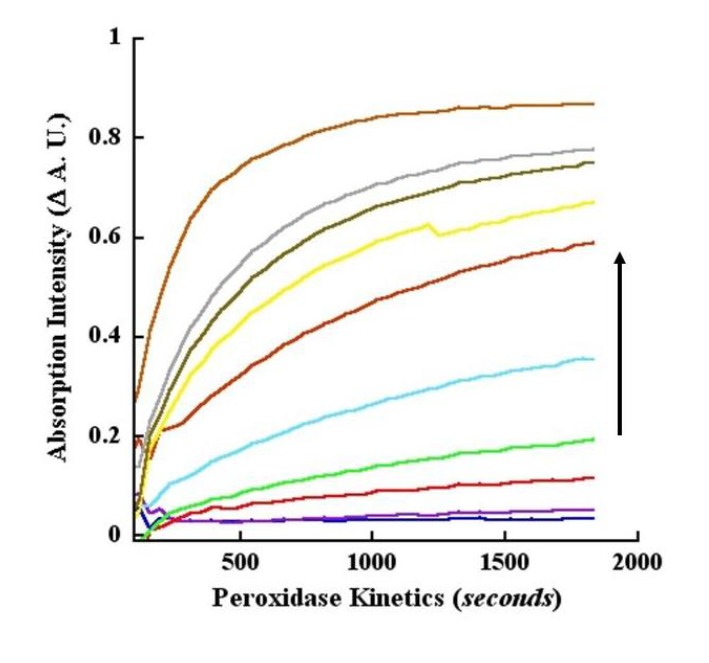
Kinetics of CA oxidation by peroxidase monitored at 295 nm. All reaction conditions were the same as described for Figure 10A, except that the data was obtained at 295 nm absorption, and transformed as B−S Δ A.U. values. Consequently, the tracings showed a progressively increasing and positive character in contrast to the declining intensities of Figure 10A. The concentrations of peroxidase represented by the various color tracings and indicated by the upward pointing arrow are the same as those shown in Figure 10A.

## 3. Experimental Section

### 3.1. Materials

Coniferyl alcohol (CA) (4-hydroxy-3-methoxycinnamyl alcohol; 4-(3-hydroxy-1-propenyl)-2-methoxyphenol; 98% pure; FW = 180.2) was purchased from Sigma Aldrich (St. Louis, MO, USA) and Alfa Aesar (Ward Hill, MA, USA) and stored at –80 °C as a 45 mg/mL solution (250 mM) in methanol. According to the certificate of analysis received from the vendors, this compound is light and air sensitive. However, when stored under conditions described above, the stock solution was stable over several months as evidenced by little or no change in its spectroscopic properties. Stock CA solution was diluted using citrate buffer, pH 4.5 just prior to use in experiments and the unused portions were discarded. Fresh dilutions of CA were prepared for each experiment. Solid CA was stored at –20 °C in a tightly closed, brown bottle as a precaution to protect from air- or light-induced oxidation. Purified laccase from *Trametes versicolor* (*T. versicolor*) was purchased from Sigma-Aldrich. To enable traceability, the details on the specific enzyme lot were provided earlier [10]. Laccase was dissolved in water and stored at –80 °C. The enzyme was also diluted in pH 4.5 buffer just prior to use as a catalyst for CA oxidation as described previously for *p*-cresol [10,11]. Streptavidin-conjugated horseradish peroxidase was from Pierce Chemical Company (Rockford, IL, USA) and used similarly to laccase. Citrate buffer, pH 4.5 was used for all experiments when spectroscopic measurements were made under acidic pH conditions. For absorption measurements under basic pH conditions, either 100 mM NaOH or 250 mM KOH was used as the final concentration; these concentrations were determined empirically as being optimum for eliciting maximal changes in absorption from CA or its oxidation products. Corning UV-transparent 96-well microplates were purchased through Fisher Scientific (Pittsburgh, PA, USA). Ultrapure water (18 MΩ∙cm) obtained from a Barnstead Nanopure water purifier (Boston, MA, USA) was used for making the solutions. All other chemicals were from commercial sources.

### 3.2. Absorption Spectroscopy of Unmodified CA

Unmodified CA was examined by absorption spectroscopy in pH 4.5 buffer or in base. Absorption measurements were carried out using 96-well UV-transparent microwell plates. All measurements were taken using a Molecular Devices M2 microwell plate reader (Sunnyvale, CA, USA) with the samples being mixed by using the “automix” function of the plate reader and/or by manually pipetting the solutions up and down. The interrogation wavelengths for absorption spectroscopy are provided in the text or in the legends to the figures. Absorption intensities are depicted on the ordinate axis of the figures as “absorption units” (A.U.) or as the difference between the signal and the background (S−B) or *vice versa* (B−S), and these differences were reported as Δ A.U. Experiment-specific details are provided in the text or in the legends to the figures.

### 3.3. Absorption Spectroscopy of Laccase-Oxidized CA

Enzymatic oxidation of CA was conducted in citrate buffer, pH 4.5 employing continuously changing catalysis time or increasing concentrations of laccase or the substrate, CA [10,11]. Enzyme aliquots were not subjected to freeze-thaw cycles and discarded after single use. The reaction mixtures consisted of the enzyme and the substrate in buffer. Reactions were interrogated in 96-well UV-transparent microplates, using various absorption wavelengths in real time kinetic (pH 4.5) or endpoint (100 mM NaOH or 250 mM KOH) mode. These changes were an index of laccase catalysis. Enzymatic and nonenzymatic reactions were carried out at controlled room temperature of ~25 °C. Absorption intensities are depicted on the ordinate axis of the figures as "absorption units" (A.U.) or as the difference between the signal and the background (S−B) or *vice versa* (B−S) and these differences were reported as Δ A.U. Experiment-specific details are provided in the text or in the legends.

### 3.4. Absorption Spectroscopy of Peroxidase-Oxidized CA

Peroxidase-catalyzed oxidation of CA was also conducted in citrate buffer, pH 4.5 similar to laccase at 25 °C. The reaction mixture consisted of peroxidase, 250 μM CA and 250 μM H_2_O_2_. Reactions were initiated by the addition of the substrate or peroxide to the other two reagents. Reaction products were interrogated in 96-well UV-transparent microplates, using various absorption wavelengths in real time kinetic measurements at pH 4.5 or in endpoint mode (100 mM NaOH or 250 mM KOH). These changes were an index of peroxidase catalysis. Absorption intensities are depicted on the ordinate axis of the figures as "absorption units" (A.U.) or as the difference between the signal and the background (S−B) or *vice versa* (B−S) and these differences were reported as Δ A.U. Experiment-specific details are provided in the text or in the legends to the figures.

### 3.5. Data Analysis

All reactions were carried out using at least triplicate measurements. Results were calculated as average ± standard deviation. Where not visible, error bars are masked within the symbol. The limits of detection (LOD) and quantitation (LOQ) were calculated as described previously [11]. All reactions incorporated several “controls” including vehicle (buffer or base) and reactions without laccase or CA. For peroxidase reactions, two additional controls were CA mixed with 250 μM H_2_O_2_ without peroxidase and CA mixed with the highest concentration of peroxidase in the absence of H_2_O_2_. The largest signal from amongst the various “control” reactions was used as the background in order to calculate the signal strength of reactions comprising the enzyme and the substrate. Kaleidagraph (v. 4.03, Synergy Software, Reading, PA, USA) was used to create the tracings shown in figures and to calculate the linearity (correlation of coefficient, *r^2^*). *EnzymeKinetics!Pro* software (v. 2.36, SynexChem LLC, Fairfield, CA, USA) was used to calculate *Km* using Lineweaver-Burk (1/V *versus* 1/S) transformation [10].

## 4. Conclusions

We describe CA detection and quantitation using optical turn on and turn off signaling in kinetic, *pseudo*-kinetic or endpoint mode under acidic or basic conditions. The same reactions may be interrogated in various modes using several different absorption wavelengths. These assays required no chemical synthesis or modification of the substrate or the product, but rather exploited the intrinsic absorption spectroscopic properties of the substrate and the product. The range of wavelengths, the hypsochromicity and bathochromicity, and the hypochromism and hyperchromism following oxidation might be a commentary on the different extents of dimerization and polymerization of CA resulting in products such as, dehydro-di-CA (β-5-dehydrodimer), *erythro*-guaiacylglycerol-β-coniferyl ether (β-O-4-dehydrodimer), *threo*-guaiacylglycerol-β-coniferyl ether (β-O-4 dehydrodimer), Pinoresinol (β-β-dehydrodimer), oligomers, and dehydrogenative polymers (DHPs) [39]. The data are in contrast to the laccase-catalyzed *p*-cresol oxidation that was accompanied by hyperchromicity only [10]. These differences might be due to the varying chemistries of the oxidation products [10,39].

Our eventual goal is a portable, low power, field deployable, multiplexed, orthogonal HTS-compatible monolignol biosensor capable of lignin compositional analyses, employing commonly used spectroscopic techniques such as absorption and fluorescence [10,11]. Such a sensor might enable the remote/standoff interrogation of biomass with respect to lignin composition, and facilitate appropriate pretreatment strategies, in order to rapidly breakdown lignin by reducing recalcitrance and improving saccharification efficiency, for producing cost-effective biofuels.
